# Pseudobulbar Affect in an Elderly Female With Small Vessel Ischemic Disease and Alcohol Abuse Disorder: A Case Report

**DOI:** 10.7759/cureus.60472

**Published:** 2024-05-16

**Authors:** Tannaz Safari, Mahdi Dehbozorgi, Bordes Laurent

**Affiliations:** 1 Neurology, American University of Antigua, St. John's, ATG; 2 Neurology, Interfaith Medical Center, Brooklyn, USA; 3 Psychiatry, American University of Antigua, St. John's, ATG

**Keywords:** emotional dysregulation, repeated mild head injury, cerebellar atrophy, corticopontine-cerebellar disinhibition, pseudobulbar palsy, alcohol abuse disorder, small vessel ischemic disease, pseudobulbar affect

## Abstract

Pseudobulbar affect (PBA) is a neurological condition characterized by recurrent, inappropriate, and involuntary outbursts of emotion, primarily crying and laughter, which are dissociated from the individual's emotional experience. The precise underlying cause of PBA remains unknown; however, existing evidence suggests the involvement of dopaminergic, serotonergic, and glutamatergic neurotransmission within the corticopontine-cerebellar pathways responsible for regulating the motor expression of emotions. Additionally, PBA has been observed to co-occur with other neurocognitive and psychiatric disorders. Therefore, it is crucial to consider the possibility of a PBA diagnosis in patients with underlying neurological damage and disorders.

## Introduction

Pseudobulbar affect (PBA) is a neurological disorder characterized by sudden, inappropriate, and involuntary bursts of laughter and/or crying, which transpire irrespective of the patient's actual mood [1]. This disorder frequently significantly impacts the patient's life, often leading to feelings of embarrassment during episodes, highlighting a dissociation between emotional expression and experience [2]. 

In recent years, comprehensive reviews of neuroimaging studies have shed light on the anatomical and neurophysiological abnormalities observed in PBA patients, with particular emphasis on the cerebellum [3,4]. It is hypothesized that pathways connecting the cortex, pons, and cerebellum play a crucial role in cognitive and affective functioning. This hypothesis finds support in the observations of patients with cerebellar lesions, who exhibit affective abnormalities and emotional lability [5]. While the exact mechanism remains unknown, it is believed that PBA results from disruptions in central serotonin, dopamine, and glutamate within corticolimbic and cerebellar pathways [2]. This disruption leads to disturbances in neurotransmitter systems involved in emotional expression and subsequent social inhibition. 

PBA can occur alongside other neurological diseases, including Alzheimer's disease, multiple sclerosis, amyotrophic lateral sclerosis, progressive supranuclear palsy, extrapyramidal and cerebellar disorders, traumatic brain injury, stroke, and brain tumors [1,2]. The prevalence of PBA in various neurological disorders has been estimated to range from 5% to 50% [6]. However, determining an exact prevalence is challenging due to differences in diagnostic criteria, methodologies, and patient populations studied. Furthermore, patients with PBA have been reported to exhibit higher rates of psychiatric illness diagnoses [2]. Consequently, PBA is frequently misdiagnosed as depression or bipolar disorder, leading to insufficient and ineffective treatment [7]. Accurate diagnosis and appropriate therapy for these patients can be facilitated through thorough assessments utilizing neuroimaging, patient history, and standardized questionnaires. 

## Case presentation

In 2022, a 66-year-old African-American female with a past medical history of hypertension, anemia, transaminitis, cataracts, and alcohol use disorder presented to the Emergency Department (ED) at Interfaith Medical Center in Brooklyn, New York, United States, complaining of fatigue and emesis. The primary medical team initially diagnosed her with acute pancreatitis but requested a psychiatric consultation due to recurrent episodes of simultaneous crying and laughing during her hospital stay. The patient reported that these episodes had been occurring for two to three years, happening multiple times per day, every day. The emotional outbursts appeared suddenly without any warning, with crying being the most frequent. The patient described her laughing outbursts as uncontrollable and unpredictable, affecting not only herself but also those around her. While she occasionally felt embarrassed by these spontaneous emotional episodes, she generally expressed fondness for them and firmly refused any treatment options offered during her admission. 

During the psychiatric interview, the patient claimed to have a consistently "great" mood and energy and expressed a strong desire to be discharged home. She denied experiencing any anxiety, helplessness, hopelessness, or any suicidal or homicidal tendencies. She also denied experiencing auditory or visual hallucinations, racing thoughts, or excessive talking. Although she mentioned having a psychiatrist, she could not recall the name and stated that she had not been diagnosed with any psychiatric illness and was not taking any psychiatric medications. The patient denied ever being hospitalized in a psychiatric ward, and there were no reported changes in her sleep or appetite. 

**Figure 1:**
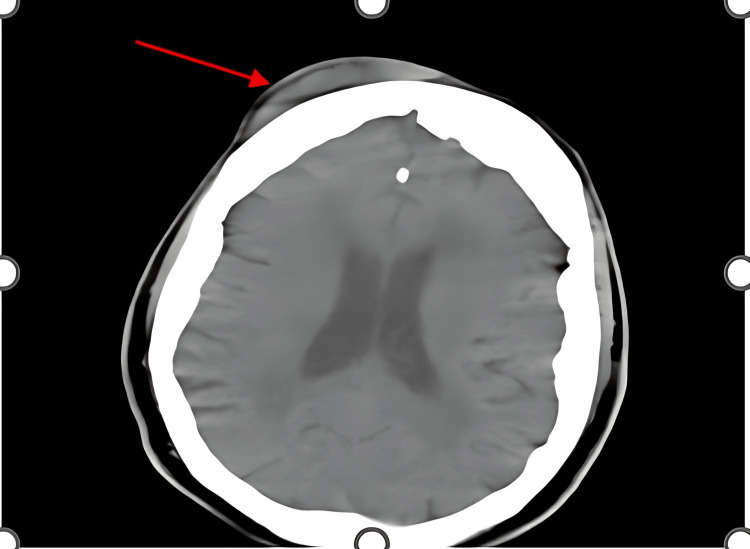
Axial CT without contrast scan of the head taken in 2020. The red arrow indicates a right frontal hematoma.

The patient disclosed a history of facial trauma 20 years ago, resulting from an altercation that led to a penetrating wound on the right side of her face. However, she denied any further history of falls, head injuries, or seizures, despite previous admission records suggesting otherwise. Her medical records indicated multiple ED admissions related to alcohol intoxication, including instances of falls and fractures. In 2018, she sustained a closed extra-articular fracture of the distal end of the right radius after a binge drinking episode during which she was pushed and fell. Another facial fracture and forehead abrasion were reported in 2020, with a CT scan without contrast of the head revealing a right frontal soft-tissue hematoma but no evidence of hydrocephalus, intra- or extra-axial blood, or other abnormalities (Figure 1).

**Figure 2:**
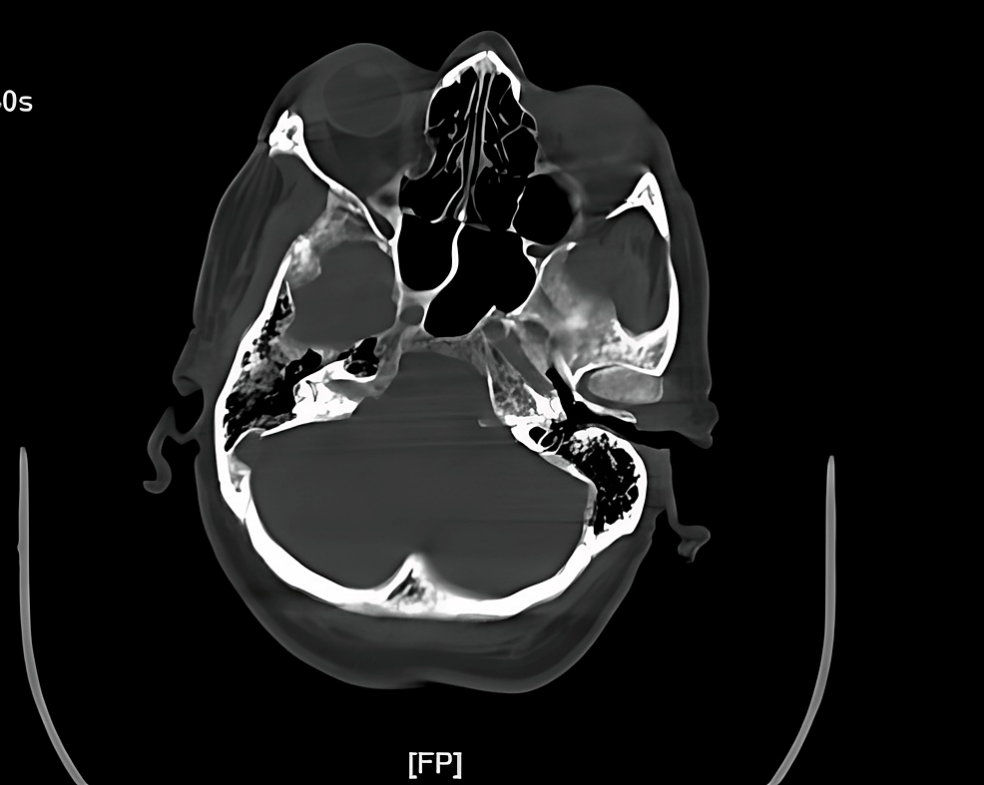
Axial CT without contrast scan of the sinuses taken in 2020.

The ventricular system was observed to be midline. Additionally, there were no focal areas of abnormal increased or decreased attenuation in either cerebral hemisphere, suggesting the absence of acute bleeding or infarction. No bony lesions were detected, and the visualized sinuses appeared normal (Figure 2). Other previous CT findings indicated age-appropriate degeneration of the lumbar spine. 

At the time of admission, the patient denied any history of tobacco, marijuana, or alcohol use. However, her previous medical records contradicted this claim, indicating multiple admissions for alcohol intoxication and frequent episodes of falling. According to her past charts, the patient consumed approximately one liter of alcohol per day and had a history of tobacco smoking for over 10 years. 

Upon examination, the patient displayed prominent emotional lability with inappropriate episodes of crying and laughing, particularly when discussing her home and past medical history or experiencing certain emotions (happiness, sadness, anger, and hunger). Prior to these episodes, lip quivering was observed. Although the patient intermittently maintained proper eye contact, she consistently exhibited tongue protrusion throughout the interaction, suggesting possible extrapyramidal signs. 

On the Mini-Mental State Exam (MMSE), the patient demonstrated orientation to person and place but not to time. She could follow commands, write her name, and spell words backward. However, she had difficulty recalling two out of three words after one minute. She successfully drew a clock and provided the correct time. Her thought process appeared linear, logical, and goal-directed, with no evidence of delusions, paranoia, or hallucinations. She denied any suicidal or homicidal thoughts, plans, or intent and demonstrated fair insight, judgment, and impulse control. Her speech was intact with normal rate, rhythm, and volume, and her cognition and concentration appeared intact overall. 

**Figure 3:**
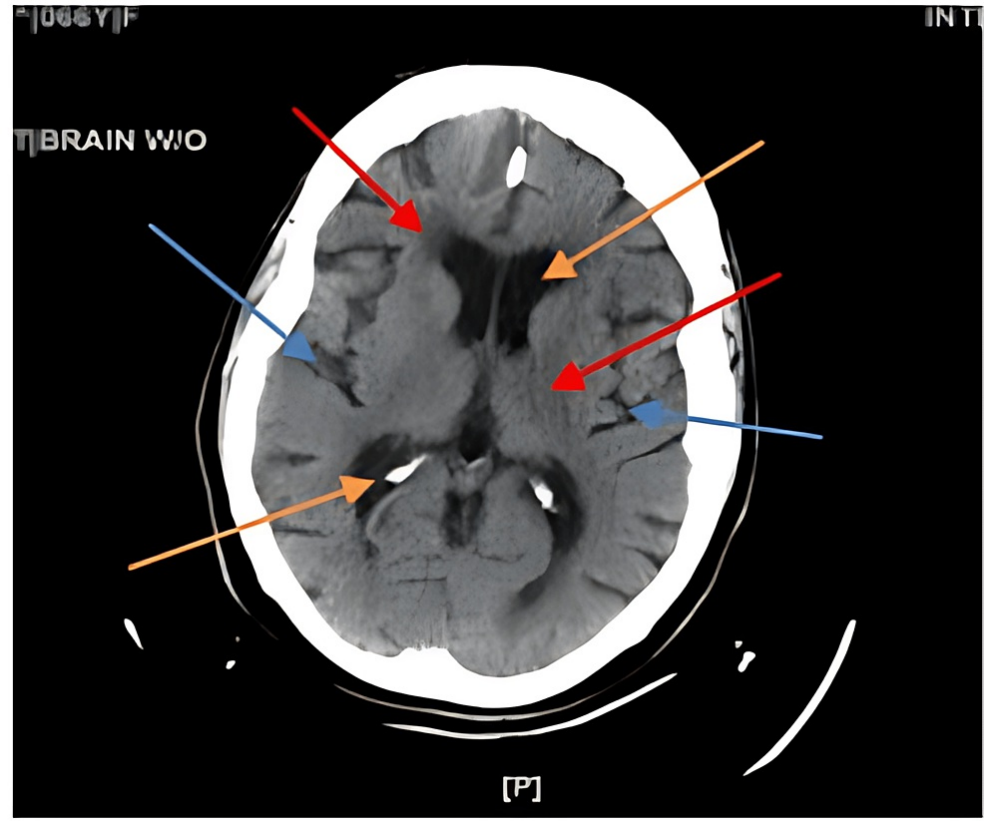
Axial CT without contrast scan of head done in 2022. Red arrows indicate areas of small vessel ischemia. Blue arrows indicate areas of volume loss. Orange arrows indicate enlarged ventricles.

**Figure 4:**
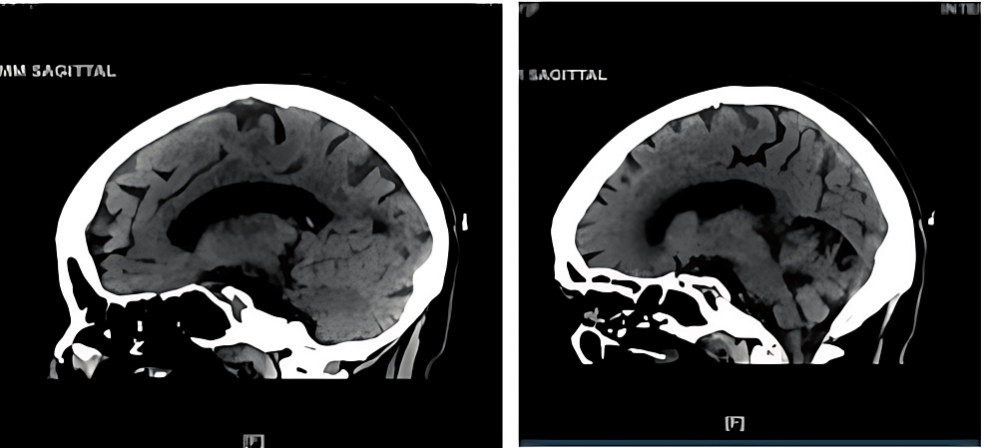
Sagittal CT without contrast scan of head done in 2022.

A CT scan without contrast of the head was conducted during her admission, revealing small vessel ischemia, volume loss without hemorrhage, and mild ventricular enlargement (Figure 3, Figure 4). Additionally, the scan identified a chronic medial right orbital wall fracture from prior trauma, mucosal thickening of the left maxillary sinus, and hardware at the anterior aspect of the right maxillary sinus. The mastoid air cells appeared normal, and no masses were detected. An abdominal CT scan showed gallstones in the common bile duct, severe liver steatosis, and renal cysts. 

Laboratory results upon admission showed a high anion gap due to alcoholic ketoacidosis, macrocytic thrombocytopenia, alkalosis resulting from dehydration caused by vomiting, asymptomatic bacteriuria, and pancytopenia with macrocytic features due to alcoholism and liver disease. The patient was initially diagnosed with starvation ketoacidosis, hypomagnesemia, dizziness, transaminitis, acute gallstone pancreatitis, and a urinary tract infection (UTI) without hematuria. Treatment included thiamine, famotidine, magnesium sulfate, and morphine sulfate. 

**Figure 5:**
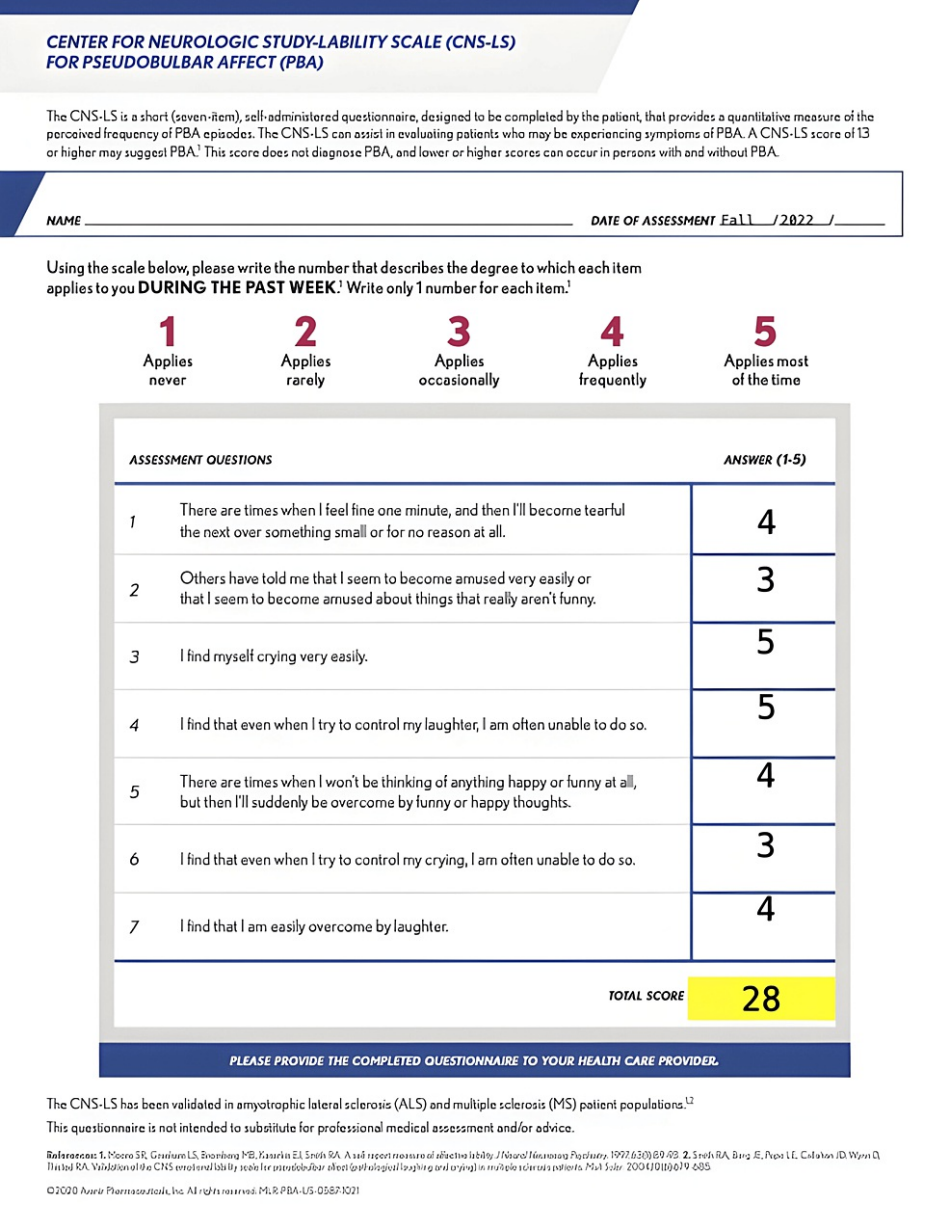
Patient score of the Center for Neurologic Study-Lability Scale Assessment used to help diagnose pseudobulbar affect in 2022.

Given the patient's history of alcohol abuse and recurrent spontaneous episodes of crying and laughter, a suspected diagnosis of PBA was considered. The Center for Neurologic Study-Lability Scale (CNS-LS) was used to assess this condition, with a cutoff score of 13 indicating a diagnosis of PBA based on previous studies [1]. The patient obtained a score of 28 on the CNS-LS survey (Figure 5). 

**Figure 6:**
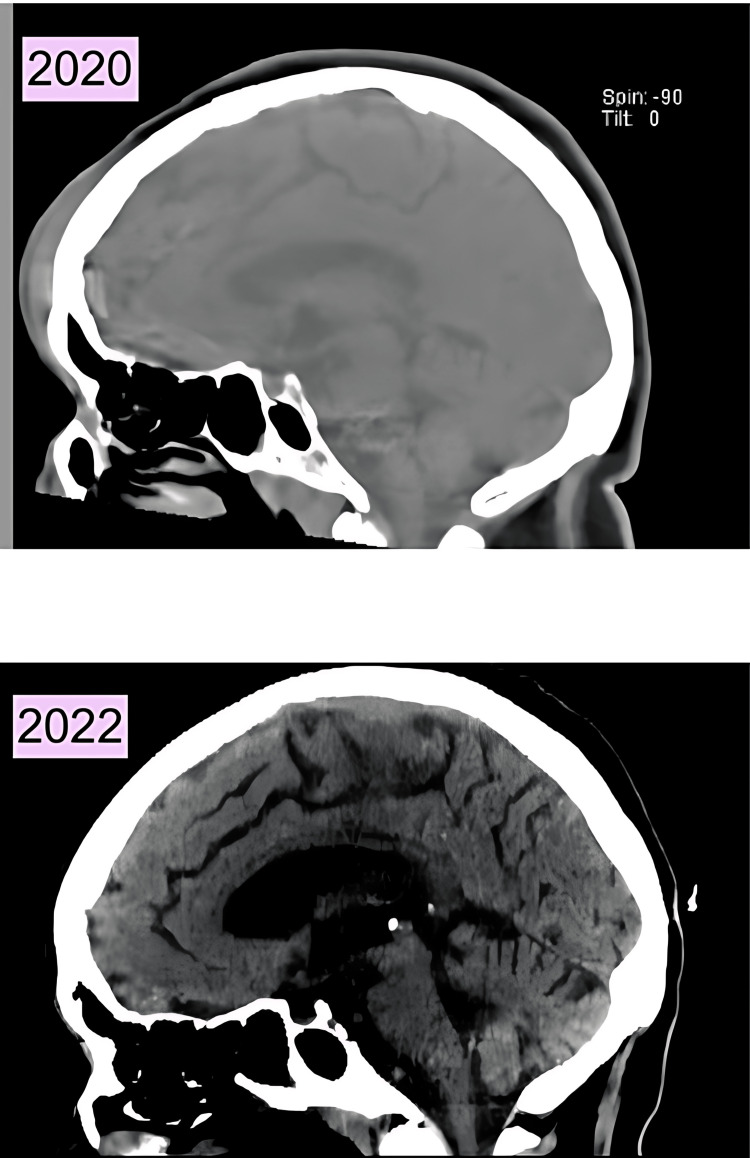
Sagittal CT without contrast scan of head done in 2022 in comparison to CT done in 2020 showing significant cerebellar atrophy.

A sagittal CT done during a 2020 admission was compared to a CT done during her current admission with the same view (Figure 6), which showed diffuse atrophy and a considerable amount of volume loss noted in the cerebellum. This is a drastic atrophy observed within a two-year time span, which could point to a potential cause of pseudobulbar palsy.

## Discussion

**Figure 7:**
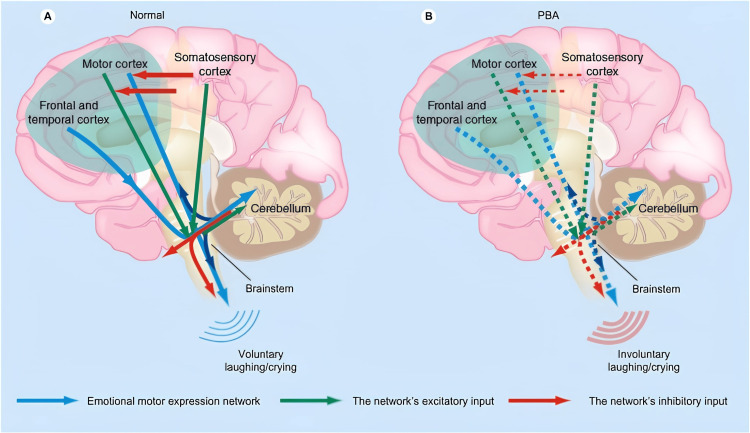
Diagram of the tracts that form the route of the cortico-ponto-cerebellar pathway Image Credit: Miller et al., 2011 [4]; Open access

This case presentation serves as an example of PBA, characterized by recurrent, involuntary, spontaneous outbursts of laughter and crying dissociated from emotional experience [1]. PBA has been linked to interactions within pathways connecting the cortex, pons, and cerebellum, primarily centered around the cerebellum as shown in Figure 7 [2]. The neuronal circuits involved in these pathways are responsible for lowering the threshold for the expression of emotions [1]. They normally function to inhibit inappropriate affect [8].

Patients with cerebellar lesions can exhibit emotional disinhibition and an inability to control emotions on cue [5]. The disruption of corticopontine-cerebellar circuits leads to impaired cerebellar modulation, resulting in PBA [2]. Both sensory and motor inputs seem to be involved in this process. A theory proposes that the cerebellum acts as a "gate control" modulating the motor control of emotions. Direct input from the motor cortex and frontal and temporal cortices through the brainstem is modulated by the cerebellum. In turn, motor input is modulated by inhibitory input from the somatosensory cortex. Reduction of the inhibitory input results in cerebellar disinhibition, leading to socially inappropriate or situationally disproportionate emotional expression observed in PBA [2].

Furthermore, evidence suggests that abnormal dopaminergic, glutamatergic, and serotonergic neurotransmission may also play a role in the development of PBA [1]. Recent neuroimaging studies have demonstrated a reduction in dopamine and serotonin expression, along with increased glutamate neurotransmission within the corticopontine-cerebellar pathways [9]. It is suggested that treatment approaches may involve increasing dopamine and serotonin levels while reducing glutamate neurotransmission [9].

Determining the risk factors and diagnosing PBA can be exceptionally challenging. The exact direct cause of PBA is unknown, but it has been observed in patients with both neurological and psychiatric disorders [2,6]. In this case, the patient had an alcohol use disorder, making cerebellar damage plausible and suggesting alcohol as a likely underlying cause of the PBA [2]. However, considering the patient's extensive history of alcohol consumption, it is possible that PBA could have resulted from a previous fall or head injury that the patient was unable to recall. The patient had a history of head trauma in 2020, resulting in a right frontal hematoma. Given the patient's history of prior trauma and alcohol abuse, it is conceivable that additional trauma may have occurred, causing damage to the cerebro-ponto-cerebellar circuit responsible for the decreased threshold of emotional expression [1].

Other potential etiologies for the patient's presentation of PBA include vascular dementia and small vessel ischemic disease [1]. The patient demonstrated cognitive impairment and possible dementia, as indicated by an inability to recall two out of three words after a minute on the MMSE. The CT imaging without contrast revealed small vessel ischemic disease. However, further investigation is needed to determine the sources of this small vessel ischemic disease. It is important to ascertain whether it is due to vascular dementia secondary to hypertension, subdural hematoma resulting from multiple falls and old age, or an extensive history of alcohol abuse. These potential causes could contribute to PBA in a multifactorial manner.

Given that the objective expression of emotion typically reflects the subjective experience of emotion, clinicians may be unaware of PBA as a distinct entity [10]. Moreover, PBA is often underdiagnosed or misdiagnosed, as it is frequently associated with other neurocognitive and psychiatric disorders [7]. Early suspicion, identification, and treatment of PBA are crucial, regardless of the underlying cause [1]. The use of validated scales such as the CNS-LS helps to objectively assess the potential diagnosis of PBA [1,9]. The CNS-LS consists of seven questions, each scored on a scale of 1 to 5, with a total possible score of 35. A score of 13 or higher on the scale suggests PBA with high sensitivity and specificity [9]. Accurate diagnosis and appropriate therapy for PBA rely on proper assessments using standardized questionnaires like the CNS-LS, neuroimaging, and patient history.

It is also important to note that while physicians have an instinct to treat identified conditions, patients may not always share the same treatment goal. In this case, our patient resisted the offered treatment options and was accepting of her PBA as it brought her joy.

## Conclusions

PBA is a neurological disorder characterized by sudden, inappropriate, and involuntary bursts of emotional expression, regardless of the patient's mood. It is often underdiagnosed or misdiagnosed due to a lack of understanding among clinicians. Although the underlying mechanisms are not fully understood, major roles are believed to be played by dopaminergic, serotonergic, and glutamatergic neurotransmission in corticopontine-cerebellar pathways. Damage to these areas within the brain may be responsible for the development of PBA. The exact relationship between PBA and neurocognitive and psychiatric disorders remains unknown, although patients with PBA often have concurrent neurological or psychiatric issues. Clinicians should be aware of PBA and its presentation, especially in patients with a history of neurological damage. The use of standardized questionnaires such as the CNS-LS, along with proper neuroimaging and patient history, should be an integral part of the differential diagnosis when a patient presents with unexplained mood affect. While both selective serotonin reuptake inhibitors (SSRIs) and tricarboxylic acids (TCAs) have been used to treat PBA, the only FDA-approved pharmacotherapy for PBA is a combination drug of dextromethorphan hydrobromide and quinine sulfate (Nuedexta). Early recognition and diagnosis of PBA by clinicians are essential for providing proper education, treatment, and awareness to improve the patient's quality of life.
